# Predicting the Malignancy Grade of Soft Tissue Sarcomas on MRI Using Conventional Image Reading and Radiomics

**DOI:** 10.3390/diagnostics14192220

**Published:** 2024-10-05

**Authors:** Fabian Schmitz, Hendrik Voigtländer, Hyungseok Jang, Heinz-Peter Schlemmer, Hans-Ulrich Kauczor, Sam Sedaghat

**Affiliations:** 1 Department of Diagnostic and Interventional Radiology, University Hospital Heidelberg, 69120 Heidelberg, Germany; fabian.schmitz@med.uni-heidelberg.de (F.S.); hendrik.voigtlaender@med.uni-heidelberg.de (H.V.); hans-ulrich.kauczor@med.uni-heidelberg.de (H.-U.K.); 2 Division of Radiology, German Cancer Research Center, 69120 Heidelberg, Germany; h.schlemmer@dkfz-heidelberg.de; 3 Department of Radiology, University of California Davis, Davis, CA 95616, USA; hyjang@ucdavis.edu

**Keywords:** soft tissue sarcoma, MRI, malignancy grade, machine learning, radiomics

## Abstract

**Objectives**: This study aims to investigate MRI features predicting the grade of STS malignancy using conventional image reading and radiomics. **Methods**: Pretherapeutic imaging data regarding size, tissue heterogeneity, peritumoral changes, necrosis, hemorrhage, and cystic degeneration were evaluated in conventional image reading. Furthermore, the tumors’ apparent diffusion coefficient (ADC) values and radiomics features were extracted and analyzed. A random forest machine learning algorithm was trained and evaluated based on the extracted features. **Results**: A total of 139 STS cases were included in this study. The mean tumor ADC and the ratio between tumor ADC to healthy muscle ADC were significantly lower in high-grade tumors (*p* = 0.001 and 0.005, respectively). Peritumoral edema (*p* < 0.001) and peritumoral contrast enhancement (*p* < 0.001) were significantly more extensive in high-grade tumors. Tumor heterogeneity was significantly increased in high-grade sarcomas, particularly in T2w- and contrast-enhanced sequences using conventional image reading (*p* < 0.001) as well as in the radiomics analysis (*p* < 0.001). Our trained random forest machine learning model predicted high-grade status with an area under the curve (AUC) of 0.97 and an F1 score of 0.93. Biopsy-underestimated tumors exhibited differences in tumor heterogeneity and peritumoral changes. **Conclusions**: Tumor heterogeneity is a key characteristic of high-grade STSs, which is discernible through conventional imaging reading and radiomics analysis. Higher STS grades are also associated with low ADC values, peritumoral edema, and peritumoral contrast enhancement.

## 1. Introduction

Soft tissue sarcomas (STSs) encompass a group of rare malignant tumors of mesenchymal origin. Reported incidences range from 1.8 to 5.0/100,000 per year, peaking at the age of 60 [1]. Age-standardized mortality rates in soft tissue sarcomas vary between 0.51 and 0.99/100,000 in men and 0.35–0.77/100,000 in women and are predicted to rise by 2025 due to insufficient advancements in STS prevention, diagnosis, and treatment [2]. Histologic grading of STSs, according to the Fédération Nationale des Centres de Lutte Contre le Cancer (FNCLCC) system, is an essential predictor for patients’ outcomes and has a crucial influence on therapy. An alternative grading system by the National Cancer Institute (NCI) is inferior to the FNCLCC in the prediction of distant metastasis and mortality [3,4]. However, histological grades from biopsy specimens can be misleading due to the heterogeneous composition of STSs [5] with consecutive adverse effects on therapy planning, especially when STS tumor grades are underestimated. Therefore, identifying imaging features capable of predicting STS grade remains a topic of ongoing discussion. Among the imaging features that are most considered useful in estimating tumor grade based on MRI are intratumoral heterogeneity, tumor margin, tumor size, peritumoral enhancement, peritumoral edema, and STS configuration/shape [6,7,8,9,10,11,12,13,14,15]. The diversity of STS subtypes adds complexity to radiological reporting, especially outside of large sarcoma centers, which increases the necessity for apparent imaging features indicating various STS specifications, such as the tumor grade. This study aims to investigate MRI features predicting the grade of STS malignancy using conventional images and radiomics. Although already some studies on radiomics and tumor grade in STSs exist [16,17,18,19,20,21,22], to the best of our knowledge, no previous study combined conventional image reading and radiomics to provide a comprehensive approach for grading soft tissue sarcomas using MRI.

## 2. Materials and Methods

### 2.1. Study Design

The institutional review board (IRB) approved this retrospective study and waived the need for written informed consent. Our radiological information system was screened for patients from the University Hospital Heidelberg and the German Cancer Research Center (DKFZ) in the period from 2013 to 2023. Patients were enrolled in this study according to the following inclusion criteria: (1) diagnosis of soft-tissue sarcoma, (2) available histologic data obtained through core biopsy, incision biopsy, or resection specimen, (3) available pretherapeutic MRI in the picture archiving and communication system (PACS). The exclusion criteria were the following: (1) lacking histologic examination, (2) missing pretherapeutic MRI imaging, (3) MRI imaging with significant artifacts, (4) primary intraosseous tumor location, (5) uterine leiomyosarcomas, (6) gastrointestinal stromal tumors. When specific MRI sequences were lacking, data were still included to analyze the other available features. All patients’ age, sex, tumor entity, and tumor grade were documented as baseline data in biopsy and/or resection specimens. Tumor grade from resected specimens was only included if the patient received no prior chemotherapy or radiotherapy. G2 and G3 tumors were summarized as high-grade STSs like in previous studies since both STS grades receive equal treatment [3,23].

### 2.2. Image Analysis

Two readers (4 and 8 years of experience) performed the image analysis with findings reached by consensus. The readers were blinded to the data and evaluated the following STS features: Tumor size was measured in three different planes, and tumor volume was calculated based on the measurements. Tumor configuration was categorized analogously to previous studies as follows: (1) ovoid/nodular, (2) fusiform, (3) multilobulated/polycyclic, or (4) streaky shape [7,24,25]. The subjective percentages of heterogenous tumor areas and ill-defined tumor margins were determined. Peritumoral enhancement and peritumoral edema were classified as (0) absent, (1) slight, (2) moderate, or (3) extensive. On the MRI slice, where tumors showed their maximum diameter, regions of interest (ROIs) were drawn on the apparent diffusions coefficient (ADC) map in a representative, solid (non-cystic and non-lipomatous) tumor component. For standardization, another ROI was drawn in the healthy muscle of the contralateral extremity. The mean ADC value of the tumor and the ratio of mean tumor ADC and the mean ADC of the healthy muscle were used for analysis. Additionally, MRI data were imported into the Mint LesionTM software (Mint Medical GmbH, Heidelberg, Germany, version 3.9.0). Using this software, ROIs were delineated around the non-lipomatous tumor component on the slices, where the tumors showed their maximum tumor diameter. In this step, T1-weighted (w), T2w, and contrast-enhanced T1w sequences (CE-T1w) were used. Radiomics features were extracted from the drawn ROIs for the subsequent statistical analysis. The radiomics analysis included 71 features per sequence and encompassed first-order statistics (such as mean intensity, maximum intensity, intensity, and histogram skewness and kurtosis, among others) and gray-level co-occurrence matrix (GLCM) features (like GLCM joint entropy, GLCM information correlation 1 and 2 or GLCM cluster tendency, among others).

### 2.3. Statistical Analysis

The collected data were analyzed using IBM SPSS statistics version 29.0.2.0. Descriptive comparisons were conducted by reporting the total amount with percentage for categorical variables and the mean with standard deviation for metric variables. Categorical variables were tested for significance using the Chi-squared and Fisher exact tests, and continuous variables were tested using Student’s *t*-test. A statistical analysis was performed to determine the differences between low-grade and high-grade STSs. An additional subgroup analysis was executed to determine the differences between low-grade STSs and underestimated high-grade STSs, which were diagnosed as grade 1 in biopsy but grade 2/3 in resection specimens. To address the multiple testing problem, q-values were calculated in Python (version 1.3.2) using the Storey method, in addition to *p*-values.

### 2.4. Machine Learning Model

Furthermore, a random forest machine learning algorithm was trained on the data acquired from the previously mentioned image analysis features using the sci-kit-learn package (version 1.3.2) in Python (version 3.12). The classifier was built with 150 decision trees. Besides that, standard hyperparameters were used without hyperparameter tuning. The model was tested on a data set based on a train test split of 0.2. The feature importance of the classifier was extracted, and the model’s performance was evaluated using the AUC-ROC score, F1 score, accuracy, specificity, and sensitivity.

## 3. Results

### 3.1. Baseline Characteristics

A total amount of 139 sarcoma cases have been eligible for this study. Among them, 40 were classified as low-grade sarcomas (G1) and 99 as high-grade sarcomas (G2 or G3). Notably, one patient developed two sarcomas: a low-grade leiomyosarcoma of the galea and a high-grade leiomyosarcoma of the lower limb. Consequently, this patient was included in the analysis with two cases. Both low-grade and high-grade sarcomas showed a higher percentage of male patients, with 70% in the G1 tumor subpopulation and 60% in the high-grade subpopulation. Mean age was lower for low-grade tumors (42.98 years ± 20.64) than high-grade tumors (57.54 years ± 28.51). In total, 5% of the patients with low-grade sarcomas developed metastasis either at the time of diagnosis or during the follow-up. In contrast, 29% of high-grade tumor patients developed metastasis. The most common sites of metastasis were multiple organs (15%), followed by solely pulmonary (10%) or hepatic exclusively (8%). The most prevalent sarcoma subtypes among low-grade patients included myxoid liposarcomas (22.5%) and myxofibrosarcomas (20%), followed by several less common sarcoma subtypes like myofibroblastic sarcomas, spindle cell sarcomas or solitary fibrous tumor which ranged between 2 and 8% in prevalence. In high-grade patients, the most common subtypes were myxofibrosarcomas (18%) followed by pleomorphic sarcomas (13%), dedifferentiated liposarcomas (13%), and synovial sarcomas (11%) among others (Table 1). Fisher’s exact test revealed a significant difference in the distribution of STS entities in both groups (*p* < 0.001). The distribution of tumor localization showed a similar pattern in both groups, with the extremities being the most common tumor site (G1: 70%, G2: 76%).

### 3.2. Conventional Imaging Features

**Figure 1:**
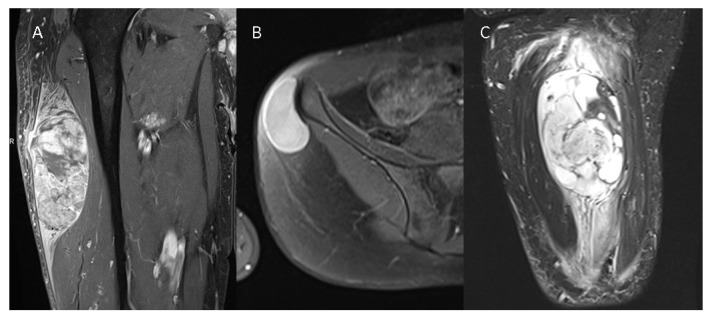
Three different STSs are illustrated—(**A**) G3 pleomorphic liposarcoma with peritumoral contrast enhancement and intratumoral heterogeneity on a contrast-enhanced T1w image, (**B**) G1 spindle cell sarcoma without peritumoral contrast enhancement and homogenous intratumoral enhancement on a contrast--enhanced T1w image, and (**C**) G2 myxofibrosarcoma of the thigh with extensive peritumoral edema on a T2 TIRM image.

High-grade tumors were significantly larger, with a mean tumor volume of 99.11 mL (±68.82 mL) in low-grade tumors compared to 127.83 mL (±65.73 mL) in high-grade tumors. While the ovoid form was the most frequently observed configuration in G1 tumors (64% of all G1 tumors), the multilobulated/polycyclic configuration was predominant in high-grade tumors, reported in approximately 73% of all high-grade cases. Tumor heterogeneity significantly varied between low-grade and high-grade STSs in T1w, T2w, and CE-T1w images (Table 2). Mean ADC values of the tumors within the ROIs were significantly higher in G1 tumors compared to G2/G3 tumors (1715 vs. 1250, *p* = 0.001). Likewise, the ratio of tumor mean ADC to healthy muscle mean ADC exhibited a significant difference (*p* = 0.005) between low-grade and high-grade STSs. Tumor margins were significantly more often less defined in high-grade tumors (T1w: *p* = 0.005, T2w: *p* = 0.002, CE-T1w: *p* = 0.009). No significant difference was observed in the extent of intratumoral hemorrhage (*p* = 0.247), cystic degeneration (*p* = 0.107), or macroscopic necrosis (*p* = 0.108, Table 2). In total, 86% of all high-grade tumors displayed intense intratumoral contrast enhancement, while 76% of the low-grade tumors did (*p* = 0.169). Peritumoral edema was significantly more severe in high-grade tumors, with 16.5% exhibiting mild peritumoral edema, 37% showing moderate peritumoral edema, and 32.99% displaying severe peritumoral edema. In contrast, 75% of all low-grade tumors demonstrated no peritumoral edema (*p* < 0.001). Similar results were observed for peritumoral contrast enhancement, with 71% of all G1 tumors displaying no peritumoral enhancement, while only 13% of all G2/G3 tumors showed no peritumoral contrast enhancement (*p* < 0.001). Figure 1 presents three different STSs.

### 3.3. Radiomics

A total of 71 texture and radiomics features were analyzed in T1w, T2w, and CE-T1w images, leading to 213 analyzed items. Sixty-nine of these items showed significant differences, as presented in Table 3. These features included CE-T1 GLCM Joint maximum (0.09 vs. 0.05; *p* = 0.03), CE-T1 Histogram Uniformity (0.11 vs. 0.07; *p* = 0.006), CE-T1 Intensity Quartile coefficient dispersion (0.13 vs. 0.20; *p* < 0.001), CE-T1 Intensity Variation (0.20 vs. 0.27; *p* < 0.001), T2 GLCM Angular second moment (0.03 vs. 0.01; *p* = 0.006) or T2 GLCM Joint entropy (55,763.15 vs. 70,230.20; *p* = 0.004), for example. No significant differences were found for T1w intensity mean (360.45 vs. 400.83, *p* = 0.725), T1w intensity max (606.92 vs. 787.67, *p* = 0.235), T2w intensity mean (512.97 vs. 476.58, *p* = 0.533), CE-T1w intensity mean (743.43 vs. 561.78, *p* = 0.225), CE-T1w intensity max (1065.20 vs. 1030.48, *p* = 0.818), T1w intensity root mean square (287,217.94 vs. 3,112,665.82, *p* = 0.521), T2w intensity root mean square (390,833.26 vs. 447,796.58, *p* = 0.25), CE-T1w intensity root mean square (383,043.31 vs. 428,679.17, *p* = 0.433) as well as intensities in the 10th, 25th, 50th, 75th, and 90th percentile for T1w, T2w, and CE-T1w.

### 3.4. Machine Learning

**Figure 2:**
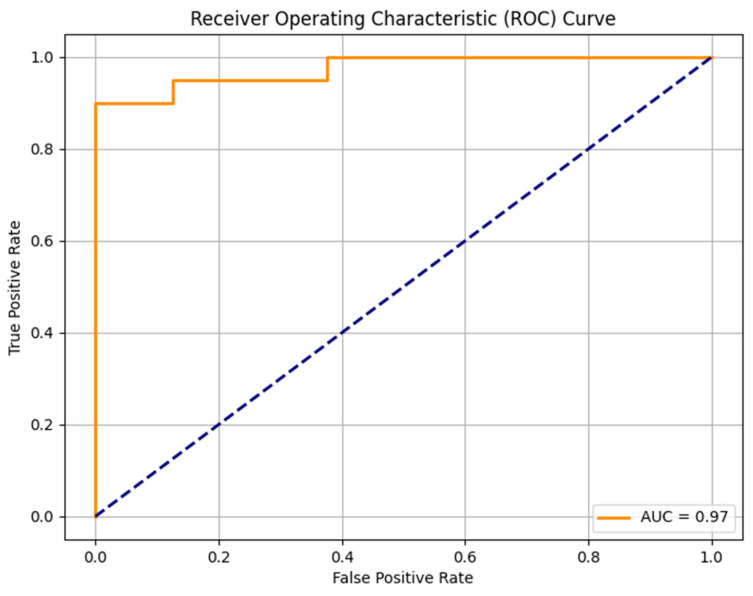
ROC curve of the random forest model to predict the STS malignancy grade.

The random forest model was trained on 111 sarcomas and tested in a set with 28 sarcomas. The model identified the most important features indicating the malignancy grades of sarcomas: intensity quartile coefficient dispersion of contrast-enhanced T1w images (feature importance 0.0357), peritumoral edema (feature importance 0.0298), heterogeneity in T2w images (feature importance 0.0247), intensity quartile coefficient dispersion in T2w images (0.0156) and histogram uniformity in T2w images (0.0133). The trained model was able to predict high-grade status in sarcomas with an AUC-ROC Score of 0.97, an F1 Score of 0.93, an accuracy of 0.89, a sensitivity of 100%, and a specificity of 62.50% (Figure 2).

### 3.5. Upgraded STS Subgroup Analysis

Six patients were initially diagnosed with FNCLCC grade 1 in a biopsy, but upon resection, they were found to have G2 sarcomas. The mean tumor volumes of G1 STSs and upgraded tumors were similar, with 99.11 mL and 95.33 mL, respectively (*p* = 0.899). Tumor heterogeneity in T2w and CE-T1w images was significantly higher in the upgraded tumors (T2w: 32% vs. 62%, *p* < 0.001; CE-T1w: 36% vs. 58%, *p* < 0.001). The difference in tumor margin was only significant in T2w images (11.5% vs. 23%, *p* = 0.045). Necrosis was almost twice as high in the upgraded tumors compared to the other G1 tumors but did not reach significance in Student’s *t*-test (*p* = 0.054). Only one patient had available diffusion imaging among the subgroup of upgraded tumors. This patient showed a lower ADC value (555) than the mean ADC from the G1 STS group (1715). Like the entire group of high-grade tumors, the upgraded tumor subgroup exhibited significant differences in peritumoral contrast enhancement and peritumoral edema compared to low-grade sarcomas (Table 2). Several radiomics features showed significant differences between definitive G1 STSs and upgraded STSs, including similar features to those in high-grade sarcomas in general, such as CE-T1 Histogram Kurtosis (*p* = 0.002), CE-T1 Intensity Mean Absolute Deviation (*p* < 0.001) or T2 GLCM Inverse Difference Moment (*p* = 0.001). The radiomics features with significant differences are listed in Table 4. Other features showed the same tendencies but without reaching significance.

## 4. Discussion

### 4.1. Tumor Size and Configuration

Tumor volume in our study was significantly larger in high-grade tumors. However, previous studies reported varying results regarding tumor volume and tumor grade [6,7,8,10,11]. These differences may arise due to variations in time until diagnosis and different growth rates in low-grade and high-grade sarcomas. In our study, the significant standard deviation reflects significant variability of tumor volume in both groups, potentially also influenced by the wide variety of tumor localizations where clinical appearance might be earlier or later. Furthermore, in our study, the difference was insignificant in q-value in multiple tests. Therefore, tumor volume is not a reliable indicator of tumor grade. Contrastingly, a better indicator of tumor grade is the tumor configuration, as already presented in earlier studies [7,25]. Our study confirmed the frequent occurrence of a multilobulated/polycyclic configuration in high-grade tumors. This may indicate rapid and irregular tumor growth and should be considered in radiological sarcoma grading.

### 4.2. Tumor Diffusion/Cellularity

A significant association of ADC values with tumor cellularity in sarcomas was proven by Schnapauff et al. [26]. Our findings reveal substantial differences in ADC means between low-grade and high-grade sarcomas most likely attributed to the high proliferative activity in high-grade tumors. G2/G3 sarcomas exhibited around 25% lower ADC values in the solid tumor component compared to G1 sarcomas. Differences based on imaging techniques were standardized using a ratio of ADC tumor mean and ADC mean of the healthy muscle. Likewise, high-grade sarcomas exhibited higher cellularity with a 36% lower ratio in high-grade STSs (1.24 vs. 0.79). Therefore, diffusion imaging provides helpful information for inferring tumor grade in STSs. The association of diffusion restriction with tumor grade has been controversial in previous literature. Some available studies found lower ADC values in high-grade STSs [10,16,24,27], while others did not [26,28,29]. However, all previous studies had smaller case numbers, so this association has been proven for the first time in a larger study cohort.

### 4.3. Peritumoral Environment and Tumor Margin

Another objective of this study has been the changes in the peritumoral environment, revealing that peritumoral edema is a characteristic feature in high-grade tumors, being present in approximately 87% of all G2/G3 tumors. In contrast, about 75% of all low-grade tumors show no peritumoral edema at all, and if present, the appearance is most likely to be mild. A prior study suggests no correlation between peritumoral edema and the histologic presence of sarcoma cells beyond the tumor margin [30]. However, for tumors in general, peritumoral immunological changes and proangiogenic behavior are recognized as caused by first growth-induced solid stress with fibroblast expression of transforming growth factor beta (TGF-β), collagen I, and alpha-smooth muscle actin, second by aberrations of fluid flow through lower interstitial fluid pressure in the peritumor compared to the tumor itself, third by tissue stiffness which can influence fibroblast and T cell function, macrophages as well as dendritic cells and fourth by distorted microarchitecture, for example, through suppression of T cell migration and infiltration due to dense matrix architecture at the tumor border with consecutive accumulation in the peritumoral tissue [31]. Furthermore, peritumoral contrast enhancement was more common and more extensive in high-grade tumors. While this could be attributed to the proangiogenic properties of the peritumor, there are also studies in other tumor entities that have shown that peritumoral contrast enhancement was associated with microvascular/lymphovascular invasion [32,33]. Ambiguous results regarding peritumoral edema and peritumoral contrast enhancement in the context of tumor grade are presented in the existing literature. Notably, especially in the larger studies, including ours, an association of peritumoral edema and peritumoral contrast enhancement with tumor grade can be shown consistently [6,11], while smaller studies failed to do so [10,34]. In our study, tumor margins were significantly less defined in high-grade sarcomas. However, the differences were minor (e.g., for CE-T1w, around 15% in G1 tumors and 23% in G2/G3 tumors), which explains why Zhao et al. and Crombe et al. were able to detect this difference as well with a study population of 95 and 130 cases, respectively [6,11], while Encinas Tobajas et al. and Gimber et al., with fewer cases, did not [28,34].

### 4.4. Hemorrhage and Necrosis

No significant differences in the extent of hemorrhage between low-grade and high-grade sarcomas were identified in conventional image reading. Our radiomics analyses reinforced the findings, which revealed no significant difference for T1w maximum intensity, T1w mean intensity, T1w intensity skewness, T1w intensity root mean square, and intensity percentiles. Similarly, the extent of confluent necrotic areas did not exhibit significant differences between both groups. Even considering that necrosis might be more extensive in larger tumors, the ratio of necrosis-to-tumor volume revealed no significant difference, which is in agreement with the results of most of the previous studies [6,8,10,29,34]. However, only a small number of previous studies found an association between the amount of necrosis and tumor grade [9,11,35]. Likewise, the intensity of contrast enhancement did not demonstrate significant differences: in both groups, most tumors showed a substantial contrast enhancement in the non-necrotic solid tumor components. This is confirmed by the radiomics analysis, where no significant difference was observed in T2w images for intensity percentiles, intensity, histogram skewness, or T2w intensity mean root square. Additionally, mean and maximum intensity in CE-T1w revealed no significant difference in contrast enhancement.

### 4.5. Tumor Heterogeneity

Tumor heterogeneity emerged in our study as a useful parameter in clinical routine: Subjective tumor heterogeneity, as assessed by the readers, was significantly higher in high-grade STSs in T1w, T2w, and CE-T1w images. However, the difference is more prominent in T2w and CE-T1w images, where the mean heterogeneity is almost twice as high in high-grade STSs. Additionally, the difference in T1w was not significant in the q-value calculated due to multiple tests. Several texture analysis features confirmed significant differences between low-grade and high-grade tumors. These include characteristic features of heterogeneity, such as histogram uniformity (a measure of how equal intensities in the histogram are distributed). This feature differed significantly in all three sequences and showed the most obvious difference in T2w (0.10 vs. 0.06, *p* = 0.001) and CE-T1w images (0.11 vs. 0.07, *p* = 0.006). In contrast, the relative difference in T1w images was more minor (0.22 vs. 0.16, *p* = 0.012). Furthermore, the histogram and intensity range were significantly higher for high-grade sarcomas in T1w and T2w images. Likewise, the intensity quartile coefficient of dispersion is higher in G2/G3 sarcomas. Kurtosis was lower in high-grade STSs in CE-T1w images, meaning a flatter distribution curve in the intensity curve and image histogram. The significant mean and median absolute deviation differences in all three sequences underline the broader distribution of intensities in high-grade sarcomas. GLCM angular second moment, a feature of image uniformity/homogeneity [36], was significantly higher in low-grade sarcomas on T2w images. Also, in CE-T1w, GLCM angular second moment was higher in low-grade sarcomas, although not reaching statistical significance. In T1w images, the relative difference was more minor but showed the same tendency. All these findings support the hypothesis that image heterogeneity, especially in T2w and CE-T1w images, helps discriminate between low-grade and high-grade STSs. T1w GLCM correlation, T1w GLCM Information correlation 1 and 2, T2w GLCM correlation, T2w GLCM information correlation 2, CE-T1w GLCM correlation, and CE-T1w GLCM information correlation were significantly higher in high-grade sarcomas which means higher correlation of intensities of neighboring voxels. Additionally, T1w GLCM inverse difference, T1w GLCM inverse, T2w inverse difference, T2w inverse difference moment normalized, and CE-T1w GLCM inverse difference moment normalized showed a lower variance of the difference between neighboring voxels. On the other hand, entropy—a measure of uncertainty [36]—was significantly higher in high-grade sarcomas in T2w and CE-T1w for T2w histogram entropy, T2w GLCM joint entropy, T2w GLCM difference entropy, and CE-T1w GLCM joint entropy. This is indicative that texture is coarser and less refined in high-grade sarcomas in our study (leading to the higher correlation of neighboring intensity values) and that uncertainty in neighboring voxels is higher in high-grade sarcomas. The theory that tumor heterogeneity is useful in predicting sarcoma grade is further underlined by the machine learning model we trained, which showed the most important feature selected by the model to be Intensity Quartile Coefficient Dispersion in CE-T1w images. In the previous literature, some uncertainty regarding the relevance of intratumoral heterogeneity exists because some smaller studies have not been able to find a significant association between tumor heterogeneity and tumor grade [10,28,29]. However, others did find an association of tumor heterogeneity in either T1w or T2w [6,8,11,18]. In the largest study to investigate this feature, we proved the relevance of intratumoral heterogeneity for inferring tumor grade in STSs. We are the first to show that tumor heterogeneity in contrast-enhanced sequences is also associated with tumor grade. Intratumoral heterogeneity results from complex genetic and molecular modifications of clonal tumor cell populations that arise in their microenvironment through Darwinian selection. This provides tumors with adaptability, for example, in hypoxia or chemotherapy, and facilitates growth and metastasis [37] and might also explain advanced dedifferentiation, elevated mitotic count, or necrosis with consequent high FNCLCC grade in highly heterogeneous tumors.

### 4.6. Machine Learning

In previous studies from the literature, the random forest classifier presented the best performance in predicting tumor grade in STSs [21,38]. Additionally, Zhang et al. showed the advantage of combining peritumoral and intratumoral features in predicting tumor grade [39]. The machine learning model we present based on a combination of intratumoral and peritumoral features and the combination of semantic imaging features and radiomics features showed a very good predictive performance and reached the highest AUC of our knowledge existing studies, even slightly better than, for example, in the study by Wang et al. [38] or the convolutional neural networks-based model by Zhang et al. [40]. Machine learning might be helpful in predicting tumor grade in STSs in the future. However, further research and development are needed to implement this in the clinical routine.

### 4.7. Upgrade

Underestimating soft tissue sarcomas in biopsy is problematic since patients miss neoadjuvant therapy. Therefore, additional suspicion through radiologic features would be helpful in this context. We found similar differences in these initially G1 graded and later found out to be G2 tumors regarding heterogeneity: Heterogeneity in T2w and CE-T1w images is significantly higher in upgraded tumors compared to definitive G1 tumors. However, for T2w, the difference was not significant anymore after Storey’s correction for multiple testing. Still, in this subgroup, radiomics features showed similar tendencies to those in the main analysis with significant differences in heterogeneity representing features in first-order statistics and GLCM especially in T2w and CE-T1w like T2w Inverse difference moment, T2w GLCM Angular second moment, T2w GLCM Joint entropy, T2w GLCM Difference entropy, T2w Histogram Uniformity or CE-T1w Intensity Mean absolute deviation. Interestingly, for the subgroup, CE-T1w GLCM cluster shade—seen as an indicator of homogeneity [36]—is significantly higher in definitive G1 tumors. A notable difference in necrosis between the two groups was observed in this study (21.32% vs. 40.00%); however, it did not reach significance (*p* = 0.054) due to the small subgroup size. A substantial portion of necrotic areas might account for the misclassification of biopsy specimens. Peritumoral enhancement and peritumoral edema are significantly more prevalent and more extensive in erroneously G1-graded tumors compared to actual G1 STSs. These findings suggest that the mentioned features could serve as valuable indicators to suspect high-grade status when FNCLCC grade 1 is misdiagnosed in biopsy specimens, which, to the best of our knowledge, has not been investigated in previous studies. Nevertheless, this aspect of our research should be investigated in further and more extensive studies.

### 4.8. Limitations

Our study has several limitations. First, as usual, all STS subtypes were analyzed together. Specific subtypes might behave differently from others. However, due to the rarity of most of the subentities in STSs, an analysis of the subtypes separately is difficult. Therefore, study populations and soft tissue sarcomas in existing literature are heterogeneously composed [41,42]. We included all STS subtypes with FNCLCC grading, including liposarcomas, extraskeletal osteosarcomas, and extraskeletal chondrosarcomas, as these were considered soft tissue sarcomas in previous studies and were also part of the original FNCLCC paper [4,43,44,45,46]. However, differences may exist between these entities in their association of imaging features with tumor grade, which should be addressed in future studies. This is particularly relevant, as different subtypes distribute differently between low and high FNCLCC grades based on their natural history [47]. Second, multiple testing problems might lead to false positive results due to the diversity of radiomics features. Third, the data were collected retrospectively. Due to the rarity of soft tissue sarcoma, prospective studies on these tumor entities are challenging and time-consuming. Fourth, our study had a single-center approach. Future studies should try a multicenter approach to evaluate our findings in larger cohorts and exclude overfitting of the model with an external validation cohort.

## 5. Conclusions

Tumor heterogeneity in T2w and CE-T1w images provides valuable information for distinguishing low-grade and high-grade sarcomas in conventional image and radiomics analyses. Additionally, alterations such as peritumoral edema, peritumoral contrast enhancement, and higher tumor cellularity support inferring the grade of malignancy in STSs. Tumor heterogeneity, peritumoral edema, and peritumoral contrast enhancement are also significantly higher in STS cases misclassified in biopsy. The trained machine learning model showed a high diagnostic performance in indicating the malignancy grade using the extracted imaging features.

## Figures and Tables

**Table 1:** Epidemiologic characteristics of the study population. SD: standard deviation.

n = 139	G1 Total Amount (Percent)/Mean (SD) n = 40	G2/G3 Total Amount (Percent)/Mean (SD) n = 99 (of Them in Biopsy Initially G1: n = 6)
Sex	Male: 28 (70%) Female: 12 (30%)	Male: 60 (60.61%) Female: 39 (39.39%)
Entity	Myxoid liposarcoma 9 (22.50%) Myxofibrosarcoma 8 (20.00%) Extraskeletal Chondrosarcoma 2 (5.00%) Synovial sarcoma 1 (2.50%) Leiomyosarcoma (extrauterine) 1 (2.50%) Spindle cell sarcoma 2 (5.00%) Myofibroblastic sarcoma/ Evans tumor 3 (7.50%) Extraskeletal Osteosarcoma 2 (5.00%) Liposarcoma NOS 2 (5.00%) Solitary fibrous tumor 2 (5.00%) Other soft tissue sarcoma 2 (5.00%) Dermatofibrosarcoma protuberans 3 (7.50%) Fibrosarcoma 3 (7.50%)	Pleomorphic sarcoma 13 (13.13%) Myxoid liposarcoma 5 (5.05%) Myxofibrosarcoma 18 (18.18%) Rhabdomyosarcoma 3 (3.03%) Pleomorphic liposarcoma 6 (6.06%) Extraskeletal Chondrosarcoma 4 (4.04%) Synovial sarcoma 11 (11.11%) Leiomyosarcoma (extrauterine) 9 (9.09%) Undifferentiated sarcoma 3 (3.03%) dedifferentiated liposarcoma 13 (13.13%) FDC-Sarcoma 1 (1.01%) MPNST 3 (3.03%) Spindle cell sarcoma 1 (1.01%) Extraskeletal Osteosarcoma 2 (2.02%) Sarcoma NOS 6 (6.06%) Other soft tissue sarcoma 1 (1.01%)
Localization	Extremity 28 (70.00%) Abdomen 4 (10.00%) Thorax 5 (12.50%) Head/Neck 3 (7.50%)	Extremity 75 (75.75%) Abdomen 19 (19.19%) Thorax 5 (5.05%)
Age	42.98 (±20.64)	57.54 (±28.51)
Presence of metastasis	(5.00%)	29 (29.29%)
Site of metastasis	Pulmonary: 2 (5.00%)	Pulmonary: 10 (10.10%) Hepatic: 8 (8.08%) Lymphatic: 1 (1.01%) Other: 2 (2.02%) Multiple 15 (15.15%)

**Table 2:** Results in conventional imaging.

	G1 STS Mean (SD)/Amount (Percentage)	G2/G3 STS Mean (SD)/Amount (Percentage)	*p*-Value (q-Value)	Upgraded to G2 Amount (Percentage/Mean (SD))	*p*-Value (q-Value)
Volume (n = 138)	99.11 mL (±68.82)	127.83 mL (±65.73)	0.023 (0.097)	95.33 mL (±55.45 mL)	0.899 (0.693)
Configuration (n = 138)	Ovoid: 25 (64.10%) Fusiform: 0 (0%) Multilobulated/polycyclic: 13 (33.33%) Streaky: 1 (2.56%))	Ovoid: 20 (20.20%) Fusiform: 4 (4.04%) Multilobulated/polycyclic: 72 (72.72%) Streaky: 3 (3.03%)	<0.001 (0.013)	Ovoid: 2 (33.3%) Fusiform: 1 (16.67%) Multilobulated/polycyclic: 3 (50%) Streaky: 0 (0%)	0.112 (0.243)
T1w Heterogeneity (reader, n = 138)	13.95% (±18.44%)	22.98% (±20.06%)	0.015 (0.073)	7.17% (±7.68%)	0.133 (0.271)
T2w Heterogeneity (reader, n = 136)	31.65% (±28.62%)	64.56% (±22.92%)	<0.001 (0.013)	62.00% (±8.37%)	<0.001 (0.013)
T1-CEw Heterogeneity (reader, n = 134)	36.11% (±26.33%)	62.39% (±23.94%)	<0.001 (0.013)	58.33% (±7.53%)	<0.001 (0.013)
T1w blurry tumor margin (n = 138)	14.98% (±20.29%)	25.36% (±19.07%)	0.005 (0.036)	27.67% (±18.62%)	0.156 (0.294)
T2w blurry tumor margin (n = 136)	11.50% (±11.69%)	20.81% (±17.30%)	0.002 (0.019)	23.00% (±12.04%)	0.045 (0.139)
T1w-CE blurry tumor margin (n = 134)	14.95% (±16.95%)	23.30% (±16.27%)	0.009 (0.049)	27.50% (±17.82%)	0.101 (0.238)
ADC mean (n = 72)	1715.79 (±555.13)	1250.10 (±402.90)	0.001 (0.013)	555.13 (-)	0.549 (0.569)
ADC mean ratio (n = 72)	1.24 (±0.51)	0.79 (±0.26)	0.005 (0.036)	0.77 (-)	0.272 (0.395)
Necrosis (n = 134)	21.32% (±25.32%)	28.74% (±23.35%)	0.108 (0.240)	40.00% (±17.89%)	0.054 (0.154)
Ratio necrosis/volume (n = 134)	0.00302 (±0.00627)	0.00301 (±0.00398)	0.993 (0.714)	0.00386	0.440 (0.520)
Extent of hemorrhage (n = 138)	4.05% (±14.42%)	7.10% (±13.81%)	0.247 (0.372)	1.00% (±0%)	0.610 (0.600)
Cystic degeneration (n = 138)	3.68% (±12.81%)	7.80% (±15.15%)	0.107 (0.240)	1.00% (±0%)	0.615 (0.600)
Intensity intratumoral contrast enhancement (n = 134)	Mild: 2 (5.26%) Moderate: 7 (18.42%) Intense: 29 (76.32)	Mild: 1 (1.04%) Moderate: 12 (12.50%) Intense: 83 (86.46%)	0.169 (0.307)	Mild: 0 (0%) Moderate: 0 (0%) Intense: 6 (100%)	0.678 (0.626)
Peritumoral contrast enhancement (n = 134)	Absent: 27 (71.05%) Mild: 8 (21.05%) Moderate: 3 (7.89%) Extensive: 0 (0%)	Absent: 14 (14.58%) Mild: 41 (42.70%) Moderate: 26 (27.08%) Extensive: 15 (15.63%)	<0.001 (0.013)	Absent: 0 (0%) Mild: 3 (50.00%) Moderate: 2 (33.33%) Extensive: 1 (16.67%)	<0.001 (0.013)
Peritumoral edema (n = 137)	Absent: 30 (75.00%) Mild: 7 (17.50%) Moderate: 2 (5.00%) Extensive: 1 (2.50%)	Absent: 13 (13.40%) Mild: 16 (16.49%) Moderate: 36 (37.11%) Extensive: 32 (32.99%)	<0.001 (0.013)	Absent: 0 (0%) Mild: 1 (16.67%) Moderate: 3 (50.00%) Extensive: 2 (33.33%)	<0.001 (0.013)

**Table 3:** Results of radiomics feature low-grade vs. high-grade.

	G1 STS Mean (SD)	G2/G3 STS Mean (SD)	*p*-Value (q-Value)
T1W Intensity Mean absolute deviation	232,006.33 (±176,002.64)	348,255.22 (±246,093.36)	0.003 (0.025)
T1W Intensity Variation	0.15 (±0.11)	0.20 (±0.10)	0.014 (0.0689
T1W Intensity Quartile coefficient dispersion	0.09 (±0.07)	0.12 (±0.07)	0.018 (0.081)
T1W Intensity Range	408.08 (±344.90)	671.86 (±780.01)	0.045 (0.139)
T1W Histogram Mean	74,133.13 (±77,912.44)	104,991.91 (±81,674.33)	0.048 (0.147)
T1W Histogram Uniformity	0.29 (±0.13)	0.16 (±0.12)	0.012 (0.063)
T1W Histogram Mean abs deviation	13,940.32 (±18,186.12)	25,217.65 (±31,537.92)	0.039 (0.135)
T1W Histogram Range	20 (±17.29)	33.15 (±38.97)	0.045 (0.139)
T1W Histogram Max	21 (±17.30)	34.15 (±38.97)	0.045 (0.139)
T1W GLCM Joint average	76,530.08 (±77,926.61)	118,705.84 (±76,443.85)	0.005 (0.036)
T1W GLCM Sum of averages	150,443.26 (±157,500.23)	232,491.27(±154,724.18)	0.007 (0.041)
T1W GLCM Sum of variance	135,382.24 (±144,996.82)	234,756.22 (±253,140.28)	0.006 (0.037)
T1W GLCM Inverse difference normalized	0.96 (±0.02)	0.98 (±0.01)	0.002 (0.019)
T1W GLCM Inverse difference moment normalized	0.99 (±0.01)	1.00 (±0.00)	0.002 (0.019)
T1W GLCM Correlation	0.73 (±0.21)	0.83 (±0.12)	0.008 (0.045)
T1W GLCM Cluster tendency	135,382.24 (±144,996.82)	234,756.22 (±253,140.28)	0.006 (0.037)
T1W GLCM Information correlation 1	−0.31 (±0.16)	−0.38 (±0.12)	0.04 (0.031)
T1W GLCM Information correlation 2	0.82 (±0.18)	0.92 (±0.08)	0.002 (0.019)
T2W Intensity Max	837.69 (±436.32)	1051.30 (±512.71)	0.025 (0.102)
T2W Intensity SD	5876.32 (±15,216.56)	658.37 (±4748.20)	0.042 (0.139)
T2W Intensity Robust mean absolute deviation	332,199.23 (±268,074.29)	445,604.28 (±302,050.45)	0.045 (0.139
T2W Intensity Median absolute deviation	79,668,561,945.33 (±170,234,845,600.41)	221,361,856,761.84 (±339,151,562,662.48)	0.002 (0.019)
T2W Intensity Variation	0.26 (±0.16)	0.34 (±0.13)	0.004 (0.031)
T2W Intensity Quartile coefficient dispersion	0.16 (±0.13)	0.22 (±0.09)	0.021 (0.090)
T2W Histogram Mean	157,639.13 (±118,809.64)	206,597.23 (±126,990.36)	0.042 (0.139)
T2W Histogram Variance	200,934.11 (±218,055.95)	345,067.15 (±276,205.08)	0.002 (0.019)
T2W Histogram Entropy	32,943.59 (±15,466.43)	43,530.46 (±13,066.41)	<0.001 (0.013)
T2W Histogram Uniformity	0.10 (±0.07)	0.06 (±0.05)	0.001 (0.013)
T2W Histogram Mean abs deviation	36,103.35 (±30,195.74)	55,749.42 (±39,606.16)	0.007 (0.041)
T2W Histogram Median abs deviation	35,049.40 (±27,708.07)	58,908.94 (±38,318.72)	0.001 (0.013)
T2W Histogram Range	31.67 (±19.99)	49.87 (±25.71)	<0.001 (0.013)
T2W Histogram Interquartile range	6.64 (±4.77)	9.91 (±6.85)	0.008 (0.046)
T2W Histogram 10th percentile	9.64 (±6.94)	12.82 (±8.11)	0.035 (0.129)
T2W Histogram 50th percentile	16.69 (±12.28)	21.52 (±12.35)	0.043 (0.139)
T2W Histogram 75th percentile	19.9 (±13.47)	26.77 (±15.10)	0.016 (0.073)
T2W Histogram 90th percentile	22.74 (±14.73)	32.32 (±17.82)	0.004 (0.031)
T2W Histogram Minimum histogram gradient intensity	19.77 (±13.08)	25.9 (±16.56)	0.042 (0.139)
T2W Histogram Max	32.67 (±19.99)	50.87 (±25.71)	<0.001 (0.013)
T2W GLCM Joint maximum	0.08 (±0.07)	0.04 (±0.05)	0.006 (0.038)
T2W GLCM Joint average	155,746.41 (±116,364.6)	214,500.28 (±126,269.28)	0.014 (0.069)
T2W GLCM Standard deviation	49,485.64 (±36,590.67)	73,024.97 (±49,559.75)	0.009 (0.049)
T2W GLCM Joint variance	231,870.78 (±247,452.02)	338,838.07 (±270,710.90)	0.036 (0.129)
T2W GLCM Joint entropy	55,763.15 (±25,922.20)	70,230.2 (±25,447.05)	0.004 (0.031)
T2W GLCM Difference entropy	21,272.38 (±9788.63)	25,060.96 (±8890.21)	0.033 (0.128)
T2W GLCM Sum of entropy	44,016.21 (±15,352.58)	50,422.99 (±17,480.11)	0.05 (0.151)
T2W GLCM Angular second moment	0.03 (±0.04)	0.01 (±0.02)	0.006 (0.037)
T2W GLCM Contrast	95,453.54 (±132,557.64)	140,697.38 (±137,282.48)	0.085 (0.216)
T2W GLCM Inverse difference	0.56 (±0.15)	0.51 (±0.13)	0.034 (0.129)
T2W GLCM Inverse difference normalized	0.95 (±0.03)	0.96 (±0.01)	0.024 (0.100)
T2W GLCM Inverse difference moment	0.51 (±0.18)	0.45 (±0.15)	0.036 (0.129)
T2W GLCM Inverse difference moment normalized	0.99 (±0.01)	1.00 (±0.00)	0.031 (0.122)
T2W GLCM Correlation	0.81 (±0.15)	0.88 (±0.08)	0.016 (0.073)
T2W GLCM Information correlation 2	0.92 (±0.07)	0.96 (±0.04)	0.014 (0.069)
CE-T1W Intensity Kurtosis	12,195.40 (±23,543.41)	956.35 (±6613.62)	0.006 (0.037)
CE-T1W Intensity Mean absolute deviation	436,541.92 (±301,950.17)	302,990.06(±276,416.66)	0.016 (0.073)
CE-T1W Intensity Variation	0.20 (±0.12)	0.27 (±0.09)	<0.001 (0.013)
CE-T1W Intensity Quartile coefficient dispersion	0.13 (±0.10)	0.20 (±0.08)	<0.001 (0.013)
CE-T1W Histogram Kurtosis	11,587.48 (±21,912.76)	1867.67 (±7245.20)	0.011 (0.060)
CE-T1W Histogram Uniformity	0.11 (±0.09)	0.07 (±0.06)	0.006 (0.037)
CE-T1W Histogram Mean abs deviation	38,465.63 (±33,889.41)	56,711.25 (±39,580.20)	0.014 (0.069)
CE-T1W Histogram Median abs deviation	38,449.87 (±33,050.33)	54,259.02 (±38,224.02)	0.028 (0.113)
CE-T1W Histogram Range	32.61 (±24.58)	44.68 (±27.20)	0.02 (0.087)
CE-T1W Histogram Interquartile range	6.79 (±5.30)	10.49 (±6.80)	0.003 (0.025)
CE-T1W Histogram 90th percentile	24.16 (±17.32)	31.69 (±19.09)	0.038 (0.133)
CE-T1W Histogram Max	33.61 (±24.58)	45.68 (±27.20)	0.02 (0.087)
CE-T1W GLCM Joint maximum	0.09 (±0.11)	0.05 (±0.07)	0.03 (0.119)
CE-T1W GLCM Joint entropy	57,339.84 (±26,233.56)	67,869.59 (±27,043.40)	0.044 (0.139)
CE-T1W GLCM Sum of averages	284,722.84 (±201,650.17)	375,143.27 (±228,733.99)	0.036 (0.129)
CE-T1W GLCM Inverse difference normalized	0.95 (±0.03)	0.96 (±0.01)	0.037 (0.131)
CE-T1W GLCM Inverse difference moment normalized	0.990474 (±0.0111258)	0.994911 (±0.0026472)	0.02 (0.087)
CE-T1W GLCM Correlation	0.78 (±0.21)	0.89 (±0.06)	0.003 (0.025)
CE-T1W GLCM Information correlation 2	0.93 (±0.07)	0.96 (±0.03)	0.016 (0.073)

**Table 4:** Results for radiomics features low-grade vs. upgraded.

	G1 STS Mean (SD)	Upgraded STSs	*p*-Value (q-Value)
T1W Intensity Variance	170,486.80 (±248,368.90)	3983.23 (±2114.30)	<0.001 (0.013)
T1W Intensity SD	16,220.97 (±21,863.11)	61.38 (±16.04)	<0.001 (0.013)
T1W Histogram Uniformity	0.29 (±0.13)	0.11 (±0.03)	<0.001 (0.013)
T1W GLCM Inverse difference moment normalized	0.99 (±0.01)	1.00 (±0.00)	<0.001 (0.013)
T1W GLCM Correlation	0.73 (±0.21)	0.89 (±0.09)	0.006 (0.037)
T1W GLCM Information correlation 2	0.82 (±0.18)	0.95 (±0.07)	0.007 (0.041)
T2W Intensity Robust mean absolute deviation	332,199.23 (±268,074.29)	737,888.40 (±227,377.83)	0.002 (0.019)
T2W Histogram Uniformity	0.10 (±0.07)	0.05 (±0.02)	<0.001 (0.013)
T2W GLCM Joint maximum	0.08 (±0.07)	0.02 (±0.01)	<0.001 (0.013)
T2W GLCM Joint entropy	55,763.15 (±25,922.20)	81,473.60 (±7811.03)	0.035 (0.129)
T2W GLCM Angular second moment	0.03 (±0.04)	0.01 (±0.00)	<0.001 (0.013)
T2W GLCM Inverse difference	0.56 (±0.15)	0.44 (±0.05)	0.002 (0.019)
T2W GLCM Inverse difference moment	0.51 (±0.18)	0.37 (±0.05)	0.001 (0.013)
T2W GLCM Information correlation 1	−0.32 (±0.12)	−0.25 (±0.02)	0.003 (0.025)
CE-T1W Intensity Mean absolute deviation	436,541.92 (±301,950.17)	97,663.99 (±80,401.06)	<0.001 (0.013)
CE-T1W Histogram Variance	232,433.75 (±233,366.82)	468,538.11 (±328,997.28)	0.035 (0.129)
CE-T1W Histogram Kurtosis	11,587.48 (±21,912.76)	0.05 (±0.6)	0.002 (0.087)
CE-T1W GLCM Joint variance	244,104.18(±221,467.32)	458,702.61 (±318,022.91)	0.044 (0.139)
CE-T1W GLCM Cluster shade	3010.86 (±330,596.43)	−343,308.54 (±387,962.95)	0.025 (0.102)

## Data Availability

Data can be made available upon reasonable request.
